# Silencing of RASSF3 by DNA Hypermethylation Is Associated with Tumorigenesis in Somatotroph Adenomas

**DOI:** 10.1371/journal.pone.0059024

**Published:** 2013-03-28

**Authors:** Hu Peng, Huanhai Liu, Shuwei Zhao, Jian Wu, Jingping Fan, Jianchun Liao

**Affiliations:** Department of Otolaryngology – Head and Neck Surgery, Changzheng Hospital, Second Military Medical University, Shanghai, China; Peking University Health Science Center, China

## Abstract

The pathogenic mechanisms underlying pituitary somatotroph adenoma formation, progression are poorly understood. To identify candidate tumor suppressor genes involved in pituitary somatotroph adenoma tumorigenesis, we used HG18 CpG plus Promoter Microarray in 27 human somatotroph adenomas and 4 normal human adenohypophyses. RASSF3 was found with frequent methylation of CpG island in its promoter region in somatotroph adenomas but rarely in adenohypophyses. This result was confirmed by pyrosequencing analysis. We also found that RASSF3 mRNA level correlated negatively to its gene promoter methylation level. RASSF3 hypermethylation and downregulation was also observed in rat GH3 and mouse GT1.1 somatotroph adenoma cell lines. 5-Aza-2′ deoxycytidine and trichostatin-A treatment induced RASSF3 promoter demethylation, and restored its expression in GH3 and GT1.1 cell lines. RASSF3 overexpression in GH3 and GT1.1 cells inhibited proliferation, induced apoptosis accompanied by increased Bax, p53, and caspase-3 protein and decreased Bcl-2 protein expression. We also found that the antitumor effect of RASSF3 was p53 dependent, and p53 knockdown blocked RASSF3-induced apoptosis and growth inhibition. Taken together, our results suggest that hypermethylation-induced RASSF3 silencing plays an important role in the tumorigenesis of pituitary somatotroph adenomas.

## Introduction

The pituitary gland regulates many functions of other endocrine glands and their target tissues throughout the body [1]. Pituitary adenomas, including somatotroph adenomas, constitute 10–15% of intracranial neoplasms [2]. Clinically detected pituitary adenomas develop in one per 10,000 persons, but present at an overall prevalence of 16.7% in the population as detected by radiology and autopsy [3]. Pituitary adenomas can cause mood disorders, sexual dysfunction, infertility, acromegaly, obesity, visual disturbances, hypertension, diabetes mellitus, and accelerated heart disease [4]. However, the pathogenic mechanisms underlying pituitary adenoma formation, progression, and invasion remain poorly understood. Mutations in classic oncogenes and tumor suppressor genes (TSGs), which might be prognostic predictors or gene therapy targets, are rarely found in pituitary tumors [1], [5]–[7]. Since the identification of RB1 TSG inactivation by promoter hypermethylation 19 years ago [8], it has become increasingly apparent that tumor suppressor promoter methylation has a significant role in the clonal evolution of cancer [9]. Indeed, several important TSGs, such as RB1 [10], FGFR2 [11], GSTP1 [12], RASSF1A [13], H-cadherin and E-cadherin [14] are rarely mutated but frequently inactivated by promoter hypermethylation in pituitary adenomas. Previously, few whole-genome methylation detection strategies have been applied to the analysis of DNA methylation in human pituitary adenomas.

To study the candidate oncogenes and TSGs involved in the pathogenesis of pituitary adenomas, we selected somatotroph adenomas, one of the most common types of pituitary adenomas [15], as representative of pituitary adenomas. We have used MeDIP (Methylated DNA immunoprecipitation) with comparative high-density whole-genome microarray analysis to identify differentially methylated regions in DNA of 27 human somatotroph adenomas and 4 normal human adenohypophyses. RASSF3, the smallest member of the RASSF family, had frequent methylation of CpG islands in its promoter regions in somatotroph adenomas but rarely in normal adenohypophyses. Promoter hypermethylation induced silencing of RASSF1A, the fist member of the RASSF family, is an early and widespread event in many tumors [13], [16]–[22]. Other members of the RASSF family, such as RASSF2A, RASSF5, RASSF6, RASSF7, and RASSF10, also show frequent DNA methylation in some tumors [23]–[25]. RASSF3 is considered to be responsible in part for resistance to mammary tumor development in neu transgenic mice [26]. However, to the best of our knowledge, there have been no previous studies about the methylation status of RASSF3 in any tumor.

To understand the relation between RASSF3 methylation and somatotroph adenoma tumorigenesis, we studied RASSF3 methylation status by DNA bisulfite treatment and pyrosequencing analysis in human adenohypophyses and somatotroph adenomas. Promoter hypermethylation of the RASSF3 gene correlated with downregulation of mRNA expression in human somatotroph adenomas. In somatotroph adenoma cell lines rat GH3 and mouse GT1.1, promoter hypermethylation and loss of RASSF3 was also observed compared to rat or mouse normal adenohypophyses. 5-Aza-2′ deoxycytidine (5-Aza) and trichostatin-A (TSA) treatment induced RASSF3 promoter demethylation, and restored its expression in GH3 and GT1.1 cell lines. To understand further the function of RASSF3, GH3 and GT1.1 cells were stably transfected with ectogenic RASSF3 by lentivirus-mediated transfection. We found that RASSF3 overexpression in GH3 and GT1.1 cells inhibited proliferation and induced apoptosis, accompanied by increased Bax, p53, and caspase-3 and decreased Bcl-2 protein expression. We also found that the antitumor effect of RASSF3 was p53 dependent, and p53 knockdown blocked RASSF3-induced apoptosis and growth inhibition.

## Materials and Methods

### Ethics Statement

This study and the use of human tissue specimens were approved by the Institutional Research Ethics Committee of the Second Military Medical University, Shanghai, China. All patients provided written informed consent, and samples were collected after surgical resection.

### Human Pituitary Tissues and Adenomas

Four normal human adenohypophyses were obtained at the time of autopsy from patients with no evidence of endocrinopathy. Histological examinations were performed to exclude the possibility of incidental pathology. Fifteen invasive somatotroph adenomas and 12 noninvasive adenomas were selected from our pituitary tumor tissue bank. All of the somatotroph adenoma specimens were obtained at the time of surgery at Changzheng Hospital (Shanghai, China). The patient sources did not receive sellar irradiation before tumor resection. All of the samples were frozen in liquid nitrogen and stored at –80°C. The tumors were characterized based on the clinical, radiological, histological, and immunohistochemical features. Tumor size and invasiveness were defined on the basis of preoperative radiological studies and operative findings, and a modification of the Hardy classification [12], as follows: Grade I tumors were microadenomas (<1 cm in diameter); and Grade II tumors consisted of enclosed macroadenomas (>1 cm in diameter) with or without suprasellar extension. Both Grade I and II tumors were defined as noninvasive. Grade III tumors exhibited local invasiveness with evidence of bony destruction and tumor within the sphenoid and/or cavernous sinus (CS). Grade IV tumors demonstrated CNS/extracranial spread with or without metastases. Grade III and IV tumors were considered to be invasive. There might be small defects in the medial wall of the CS, therefore, noninvasive pituitary adenomas are able to grow into the CS through defects and show an illusion of CS invasion [14]. Therefore, CS invasion on magnetic resonance imaging was not considered a sufficient condition for invasion in the current study. Of the 27 tumors, 7 were Grade I, 5 were Grade II, 6 were Grade III, and 9 were Grade IV.

### HG18 CpG Plus Promoter Microarray

DNA samples from 27 human somatotroph adenomas and 4 normal human adenohypophyses were extracted according to the manufacturer’s instructions (NimbleGen) and sonicated to random fragments of 200–1000 bp. Immunoprecipitation of methylated DNA was performed using Biomag™ magnetic beads coupled to mouse monoclonal antibody against 5-methylcytidine. The immunoprecipitated DNA was eluted and purified by phenol-chloroform extraction and ethanol precipitation. The total input and immunoprecipitated DNA were labeled with Cy3- and Cy5-labeled random 9-mers, respectively, and hybridized to a NimbleGen HG18 CpG Promoter array, which is a single array design containing all known CpG islands annotated by University of California Santa Cruz (UCSC) Genome Browser and all well-characterized RefSeq promoter regions (from about –800 to +200 bp of the transcriptional start sites (TSSs) totally covered by ∼385,000 probes. Scanning was performed with the Axon GenePix 4000B microarray scanner. Raw data were extracted as pair files by NimbleScan software. Estimation of DNA methylation levels based on microarray was accomplished by the method described by Pelizzola [27].

### DNA Bisulfite Treatment and Pyrosequencing Analysis

DNA from tissues and cell lines was purified using a DNeasy Tissue Kit (Qiagen). Then DNA was bisulfite modified using the EZ DNA methylation kit (Zymo Research, Orange, CA, USA) according to the manufacturer’s recommendations. The RASSF3 promoter CpG island was identified by using UCSC CpG island online search tool (http://genome.ucsc.edu/). The pyrosequencing primers were designed using PSQ Assay Design Software (Biotage, Uppsala, Sweden). A 500-ng aliquot of modified DNA was subjected to PCR amplification of the specific promoter region using the designed primers and the Platinum PCR SuperMix High Fidelity Enzyme Mix (Invitrogen, Carlsbad, CA, USA). The PCR products were checked by gel electrophoresis to confirm the size of the product and rule out the formation of primer dimers. The specific PCR products were then subjected to quantitative pyrosequencing analysis using a Biotage PyroMark MD System (Biotage), following the manfacturer’s protocol. The results were analyzed by Pyro Q-CpG 1.0.9 software (Biotage). Statistical analysis was performed to detect significant changes in the frequencies of DNA methylation of the CpG sites between tumor and normal samples.

### Cell Lines

Rat GH3 and mouse GT1.1 somatotroph adenoma cells were obtained from the Chinese Academy of Sciences (Shanghai, China). Cells were maintained in Dulbecco’s Modified Eagle’s Medium (DMEM)/F12+10% fetal bovine serum (FBS) supplemented with 100 U/ml penicillin and 100 U/ml streptomycin, and incubated at 37°C in 5% CO_2_.

### Quantitative Real-time Reverse Transcriptase PCR (qRT-PCR) Analysis of RASSF3

Total RNA was isolated using the RNeasy Mini-kit (Qiagen, Valencia, CA, USA). Single-stranded cDNA was subsequently synthesized using the iScript cDNA Synthesis Kit (Bio-Rad, Hercules, CA, USA). Expression of RASSF3 was investigated in human adenohypophyses and somatotroph adenomas, rat and mouse adenohypophyses, GH3 and GT1.1 cells. The RASSF3 primers were designed using the online software, Primer 3 (http://frodo.wi.mit.edu/). qRT-PCR was performed using an iCycler (Bio-Rad) with the threshold cycle number determined by iCycler software, version 3.0. Results for the RASSF3 gene were normalized to β-actin gene, which had minimal variation in all normal and tumor samples tested, and is therefore considered to be a reliable and stable reference gene for RT-PCR.

### 5-Aza and TSA Treatment

For validation of the role of promoter DNA hypermethylation in transcriptional regulation of RASSF3 *in vitro*, somatotroph adenomas cell lines GH3 and GT1.1 were used. GH3 and GT1.1 cells were maintained in DMEM supplemented with 10% FBS and antibiotics (Invitrogen). Cells were seeded at low density for 24 h and then treated with 4 mM 5-Aza (Sigma-Aldrich, St. Louis, MO, USA) for 72 h or 250 nM TSA (Wako, Osaka, Japan) for 24 h. Total RNA and DNA were isolated and purified by RNeasy and DNeasy tissue kits (Qiagen). DNA methylation levels of the CpG nucleotides of the RASSF3 promoter were determined by pyrosequencing. The RASSF3 mRNA expression levels were determined by qRT-PCR.

### RASSF3 and p53 Overexpression and Silencing in GH3 and GT1.1 Cells

Rat GH3 and mouse GT1.1 somatotroph adenoma cells were obtained from the Chinese Academy of Sciences (Shanghai, China). Cells were maintained in DMEM/F12+10% FBS supplemented with 100 U/ml penicillin and 100 U/ml streptomycin, and incubated at 37°C in 5% CO_2_. A full-length human RASSF3 cDNA and RASSF3 siRNA were subcloned into the pLVTHM lentivirus vector (Invitrogen) to construct Lenti-RASSF3 and Lenti-RASSF3 shRNA. Conditioned medium containing lentiviruses was harvested 48 h after transfection of HEK293T cells. This medium was filtered and used to infect recipient cells in the presence of 10 µg/ml polybrene. Transfection of Lenti-p53 and Lenti-p53 shRNA was similar to RASSF3.

### Protein Extraction and Western Blotting

Proteins were extracted from subconfluent cultures and subjected to western blot analysis. After blocking with 5% nonfat milk in phosphate-buffered saline with Tween (PBS-T) for 1 h at room temperature, the membranes (Protran; Schleicher & Schuell, Dassel, Germany) were blotted with primary antibody, followed by incubation with a peroxidase-conjugated secondary antibody. Bound antibodies were visualized using enhanced chemiluminescence (Bio-Rad, Richmond, CA, USA). The primary antibodies used were as follows: rabbit polyclonal antibody to RASSF3; mouse monoclonal antibody to p53; goat polyclonal antibody to BAX; goat polyclonal antibody to caspase-3; mouse monoclonal antibody to Bcl-2; goat polyclonal antibody to matrix metalloproteinase (MMP)-2; mouse monoclonal antibody to MMP-9; and a rabbit polyclonal antibody to β-actin used as a gel loading control. These antibodies were purchased from Santa Cruz Biotechnology (Santa Cruz, CA, USA).

### Cell Proliferation Assay

Cell growth was determined by the 3-(4,5-dimethylthiazol-2-yl)-2,5-diphenyltetrazolium bromide (MTT) colorimetric assay. Nontransfected GH3 and GT1.1 cells and stably transfected cells (Lenti-RASSF3, Lenti-RASSF3 shRNA, and Lenti-GFP) were replated onto 96-well plates at 4×10^3^ cells/well and cultured overnight to allow for attachment. At daily intervals (24, 48, 72, 96, and 120 h), the number of viable cells was determined by MTT assay. Cells were incubated with 0.2 µg/ml MTT for 4 h in the dark at 37°C. After removal of the medium, the formazan crystals produced from MTT by live cells were dissolved in 150 µl dimethylsulfoxide (DMSO), and absorbance was measured at 570 nm with an Ultra Multifunctional Microplate Reader (Tecan, Durham, NC, USA). Three independent experiments were performed in quadruplicate wells.

### Apoptosis Assay

Apoptosis was measured using an Annexin V/propidium iodide (PI) apoptosis detection kit (Bender MedSystem, Vienna, Austria). Cells cultured in 6-cm dishes were trypsinized, washed, stained with PI-conjugated anti-Annexin V antibody in the dark for 15 min at room temperature, and analyzed by flow cytometry (FACSCalibur; Becton–Dickinson, Mountain View, CA, USA).

### Invasion Assay

An equal number (1×10^5^) of nontransfected cells, as well as cells stably transfected with Lenti-RASSF3 shRNA, Lenti-RASSF3, or Lenti-GFP, were plated onto separate 24-well cell culture inserts coated with Matrigel with 8-µm pores. Minimum essential medium with 10% FBS was added to the lower chamber as a chemoattractant. After 24 h incubation at 37°C in a 5% CO_2_ atmosphere, cells remaining adherent to the upper surface of the filter were removed using a cotton applicator. The cells on the lower surface of the membrane (migrated cells) were fixed with 3.7% formaldehyde, stained with hematoxylin, and counted. The invasion rate was determined from three independent experiments.

### Statistical Analysis

GraphPad Prism software version 5.0 (La Jolla, CA, USA) was used for all of the statistical analyses. All experimental data are expressed as the mean ± standard deviation of three independent experiments. Statistical analysis between the two groups was performed using Student’s *t*-test (unpaired *t*-test). The correlation between the DNA methylation level and mRNA expression was determined by Spearman’s rank correlation. All P values were based on two-tailed tests and differences were considered statistically significant when the P value was <0.5.

## Results

### RASSF3 Promoter Methylation is Higher in Human Somatotroph Adenomas in HG18 CpG Plus Promoter Microarray

**Figure 1:**
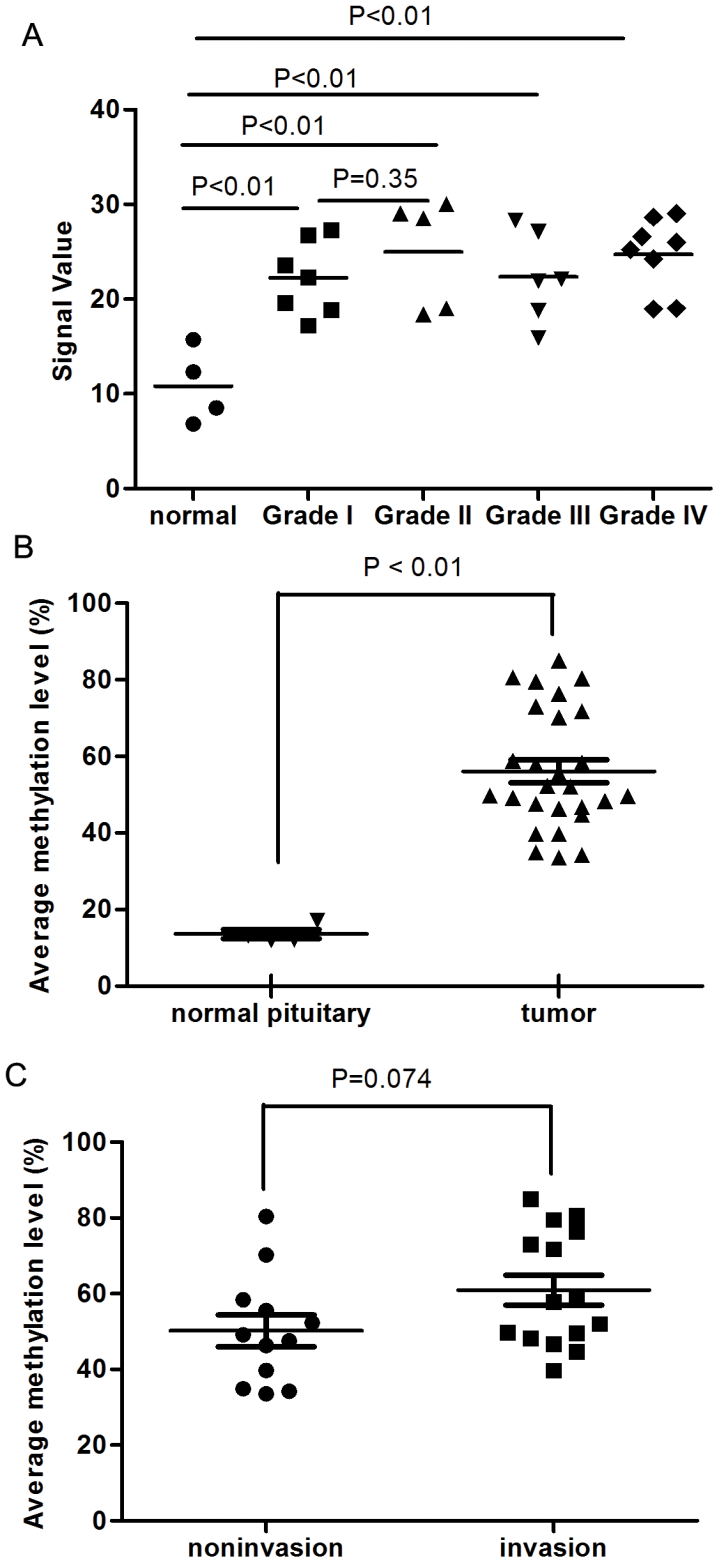
RASSF3 is hypermethylated in pituitary adenomas. (A) Average signal value in RASSF3 promoter of 27 tumors and 4 adenohypophyses. Signal values of the probes were graded from 1 to 32. The average signal values in every grade of tumor were significantly higher than in 4 adenohypophyses. However, there was no significant difference between different tumor grades. (B) Pyrosequencing technology demomstrates that RASSF3 promoter methylation level is higher in human somatotroph adenomas (P<0.01). The average methylation level of the 10 CpG sites in RASSF3 promoter of the 4 normal samples and 27 tumor samples is shown.

We used MeDIP with comparative high-density whole-genome microarray analysis to identify differentially methylated regions in DNA from 27 human somatotroph adenomas and 4 normal human adenohypophyses. Average methylation signal values of the 15 probes position in RASSF3 promoter of the 31 samples are shown in Figure 1A. The average value in 27 tumors was significantly higher than that in the 4 adenohypophyses. However, there was no significant difference between tumor grades.

To confirm the methylation level in RASSF3, we used a quantitative technology – pyrosequencing. One CpG island was identified in the human RASSF3 promoter region. Ten CpG sites in the CpG island were selected to represent the methylation status of the RASSF3 promoter region. The average methylation level for the RASSF3 gene promoter determined by pyrosequencing technology in human somatotroph adenomas was significantly higher than that in normal human adenohypophyses (Figure 1B). However, there was no significant correlation between RASSF3 methylation and tumor grade (Figure 1C).

### Downregulation of RASSF3 mRNA Expression Correlates with Promoter Hypermethylation in Human Somatotroph Adenomas

**Figure 2:**
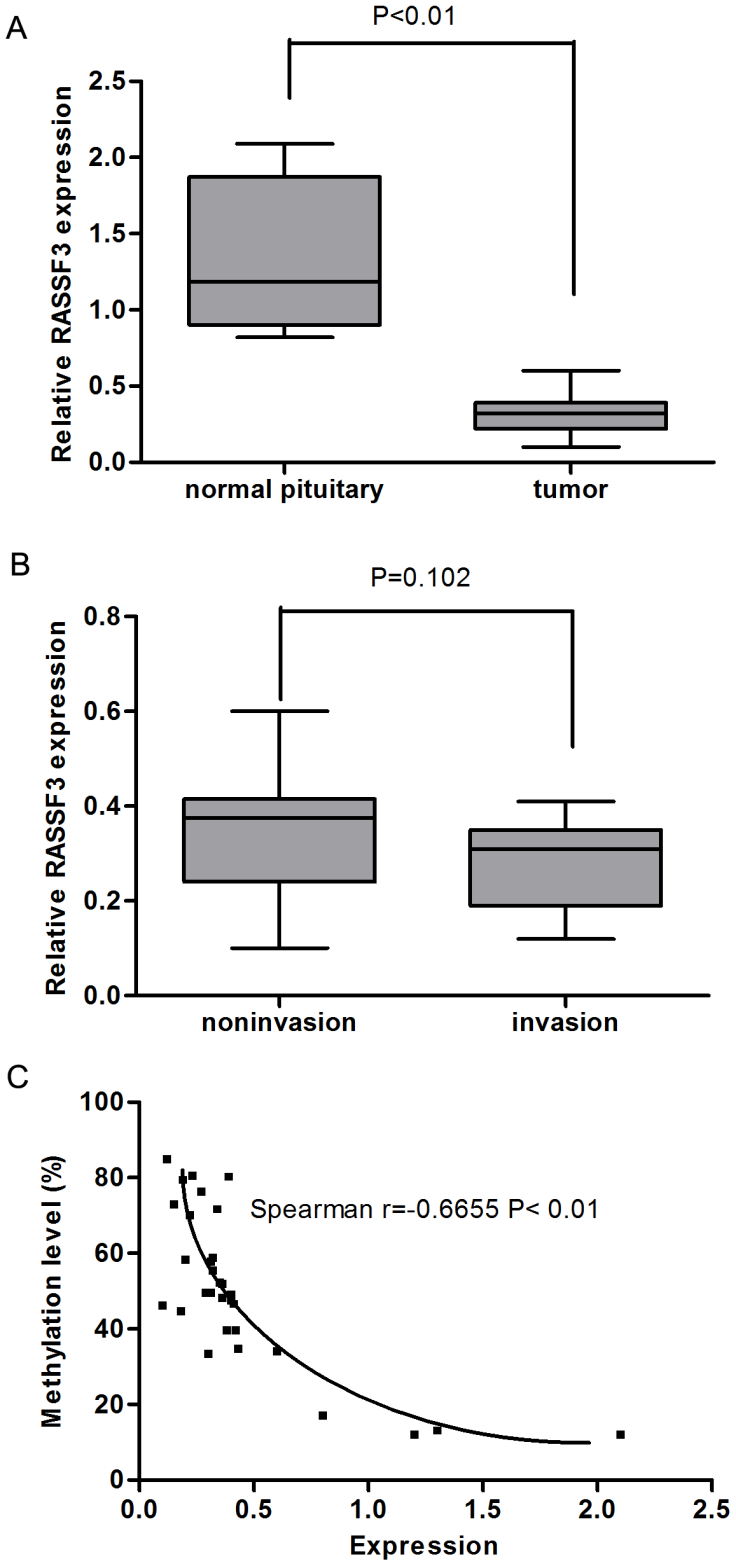
RASSF3 downregulation correlates with its promoter hypermethylation in human somatotroph adenomas. (A) The average relative RASSF3 mRNA level in 4 normal samples and 27 tumor samples is shown. RASSF3 expression level was significantly lower in human somatotroph adenomas than in normal pituitary samples (P<0.01). (B) There was no significant difference in RASSF3 methylation and expression levels between different tumor grades. (C) Spearman analysis of all normal and tumor samples with RASSF3 mRNA level and promoter region methylation level demonstrated a significant inverse correlation (*r* = –0.6655) between DNA hypermethylation and gene expression.

We next investigated RASSF3 mRNA level in the 27 pituitary adenomas and 4 normal pituitary tissues by qRT-PCR. RASSF3 expression level was significantly lower in human somatotroph adenomas than in normal adenohypophyses (Figure 2A). However, the reduced expression of RASSF3 was not related to tumor grade (Figure 2B). Using the Spearman rank correlation, we found a significant inverse correlation between promoter methylation and mRNA expression of RASSF3 (Figure 2C). These results suggest that the hypermethylation of the RASSF3 promoter region might be the reason for the suppression of its mRNA expression in human somatotroph adenomas.

### Promoter Hypermethylation of the RASSF3 Gene and Downregulation of mRNA Expression is also Observed in Somatotroph Adenoma Cell Lines

To gain an insight into the contribution of gene promoter methylation toward aberrant expression of RASSF3, we studied RASSF3 methylation status and its expression level in normal rat and mouse normal adenohypophyses, and mouse GT1.1 and rat GH3 tumor cell lines. *In silico* data analysis of the mouse and rat RASSF3 genes identified *bona fide* CpG islands in the rat and mouse homolog of the RASSF3 gene. In both rat and mouse tumors, the RASSF3 gene had a single CpG island in the presumed promoter region. Ten CpG sites in the CpG island were selected to represent the methylation status of RASSF3 promoter region in both rat and mouse tumors. The average methylation level in rat and mouse normal adenohypophyses was 14% and 12%, respectively. Methylation level of the RASSF3 promoter in rat GH3 and mouse GT1.1 cells was 78% and 85%, respectively (Table 1). We also investigated RASSF3 expression level in somatotroph adenoma cell lines and rat and mouse normal adenohypophyses. As shown in Table 1, significantly lower levels of RASSF3 mRNA were observed in GH3 and GT1.1 compared with normal adenohypophyses.

**Table 1:** Methylation and expression of RASSF3 in rat and mouse normal pituitary samples and somatotroph adenoma cell lines.

Sample	Methylation (%)	Expression
Rat pituitary	14	1.6
GH3	85	0.1
Mouse pituitary	12	1.8
GT1.1	78	0.2

Methylation levels were investigated using pyrosequencing and are shown as an average level of the 10 CpG sites in the RASSF3 promoter region. RASSF3 mRNA expression fold was determined using qRT-PCR, and β-actin mRNA level was also analyzed as endogenous control. In both GH3 and GT1.1 cells, RASSF3 methylation level was higher and mRNA level was lower than in normal pituitary samples.

### 5-Aza and TSA Treatment Restores RASSF3 Expression in Silenced Somatotroph Adenoma Cell Lines

**Figure 3:**
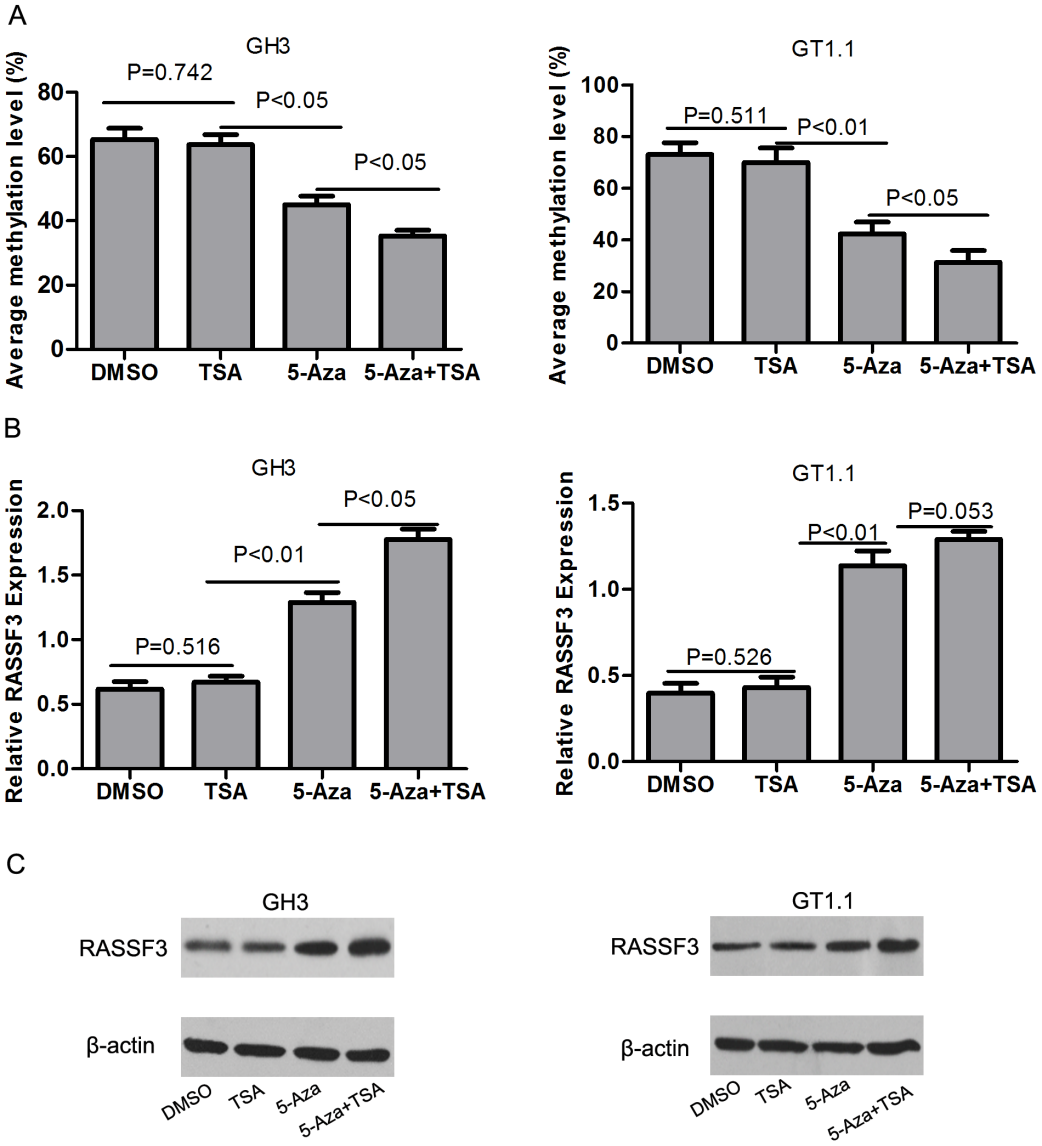
Demethylation treatment increased RASSF3 gene expression in somatotroph adenoma cell lines with RASSF3 hypomethylation. (A) DNA methylation levels of corresponding samples are shown. 5-Aza treatment induced RASSF3 demethylation, and administration of TSA following 5-Aza led to a further decrease in the methylation level of RASSF3. (B) Relative GT1.1 expression ratios normalized to β-actin are shown. Decreased methylation and increased mRNA level of RASSF3 were observed after 5-Aza treatment. In GH3 cells, TSA treatment after 5-Aza had a significant additive effect in restoring RASSF3 expression. This additive effect of TSA was also observed in GT1.1 cells, athough it was not significant. (C) RASSF3 protein level was increased after 5-Aza and TSA treatment.

To establish whether methylation of RASSF3 was the reason for its silencing, we examined the impact of the methylation inhibitor **5-Aza** and histone deacetylase inhibitor TSA on GT1.1 and GH3 cells. As shown in Figure 3A and B, in GT1.1 and GH3 cell lines, 5-Aza treatment induced RASSF3 mRNA re-expression, and this was associated with promoter demethylation. TSA treatment alone did not restore RASSF3 expression or alter its methylation levels. However, administration of TSA following 5-Aza had an additive effect in restoring RASSF3 expression in GH3 cells. Furthermore, TSA treatment after 5-Aza led to a further decrease in the methylation level of RASSF3. To confirm that RASSF3 protein level was restored after 5-Aza treatment, we investigated RASSF3 level by western blotting in GT1.1 and GH3 cells. As shown in Figure 3C, there was a significant increase in the RASSF3 protein level after 5-Aza treatment as compared to DMSO, and TSA treatment after 5-Aza had an additive effect on RASSF3 protein level. Western blotting suggested that 5-Aza and 5-Aza+TSA can restore the RASSF3 protein expression in these cells.

### Overexpression of RASSF3 Inhibits Growth and Induces Apoptosis in Somatotroph Adenoma Cell Lines

**Figure 4:**
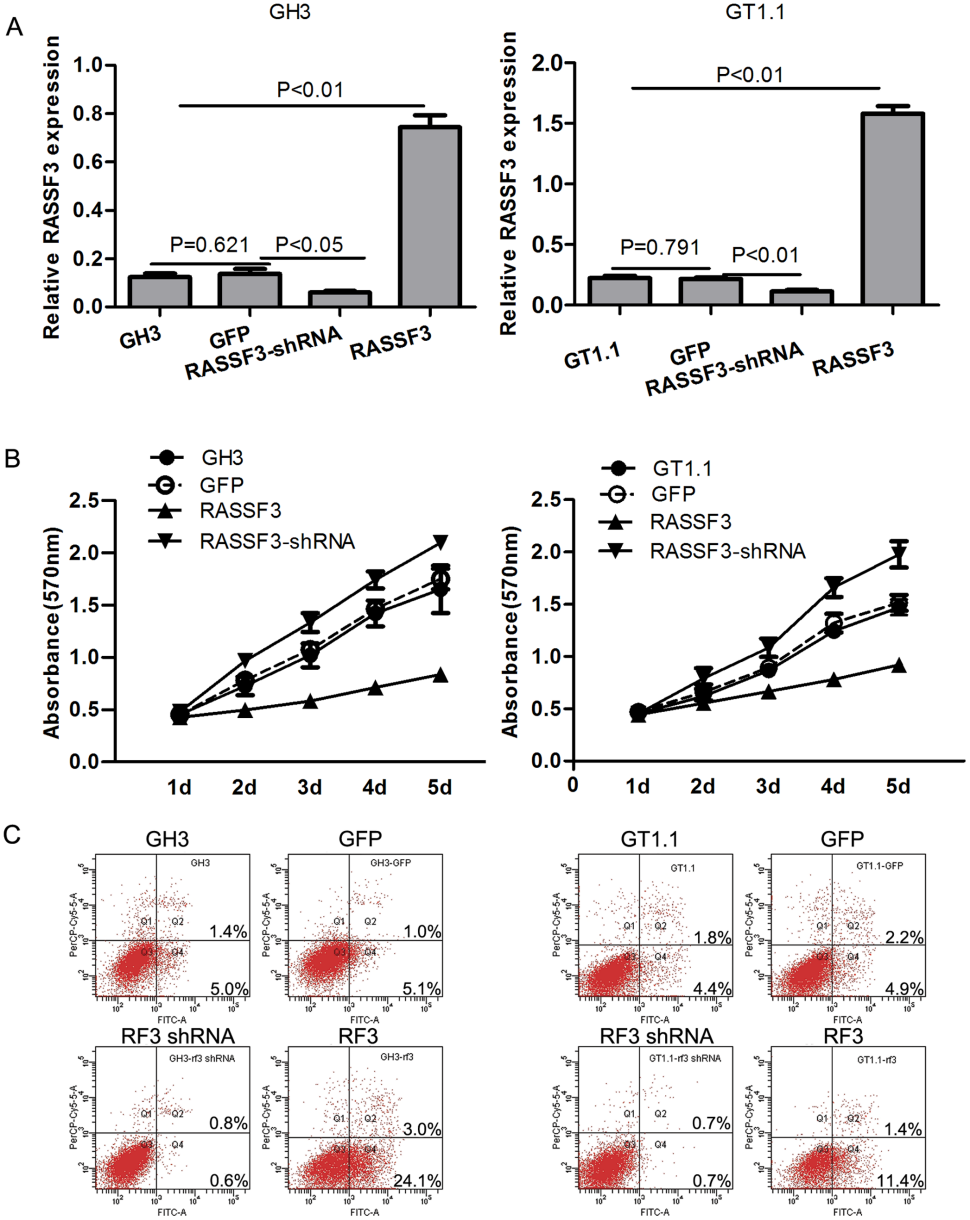
Effects of RASSF3 transfection on somatotroph cell proliferation and apoptosis. (A) Compared with cells stably transfected with Lenti-GFP, qRT-PCR analysis demonstrated that RASSF3 mRNA was significantly improved in somatotroph cells stably transfected with Lenti-RASSF3, and decreased in somatotroph cells stably transfected with Lenti-RASSF3 shRNA. (B) Effect of RASSF3 overexpression and suppression on GH3 cell proliferation was measured by MTT assay. Absorbance was read at 570 nm and the average value was calculated from 3 wells. (P<0.05 at 48, 72, 96, and 120 h for Lenti-RASSF3 or Lenti-RASSF3 shRNA versus Lenti-GFP or nontransfected GH3 cells). The results in GT1.1 cells were similar to GH3 cells. (C) Apoptosis in GH3 cells was measured by Annexin V/PI staining following Lenti-RASSF3 transfection. Early apoptotic cell populations were significantly increased after Lenti-RASSF3 transfection, and decreased after Lenti-RASSF3 shRNA transfection. The results in GT1.1 cells were similar to GH3 cells. RF3: abbreviation of RASSF3.

**Figure 5:**
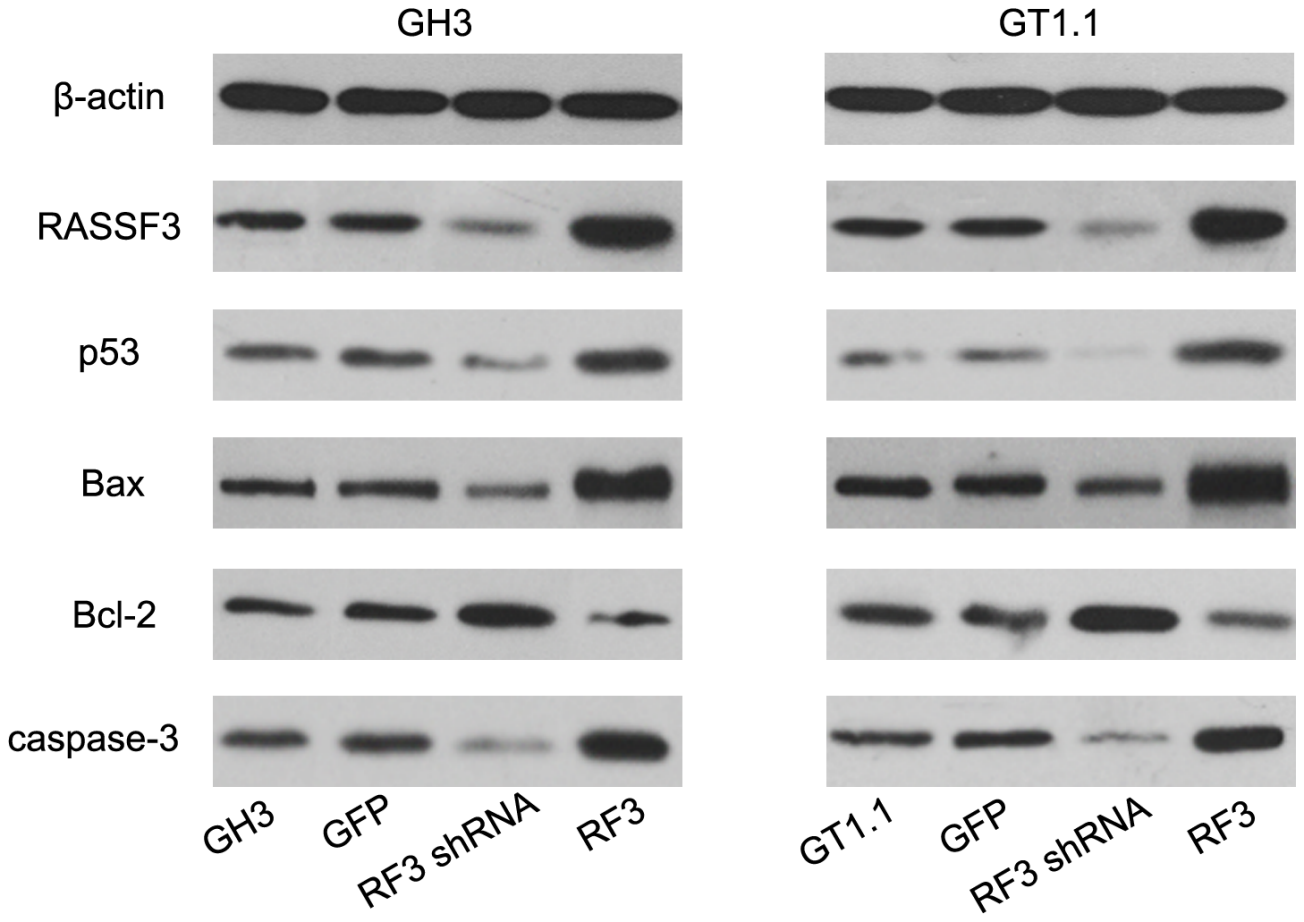
Western blotting analysis for RASSF3, p53, Bax, Bcl-2, and caspase-3. β-actin served as an endogenous control. Increased expression of p53, Bax, and caspase-3 and decreased expression of Bcl-2 were observed in GH3 and GT1.1 cells 72 h after Lenti-RASSF3 transfection as compared with the control groups (P<0.05).

To understand the function of RASSF3 in pituitary adenomas, we overexpressed and knocked down RASSF3 in GH3 and GT1.1 cells. After transfection, qRT-PCR analysis for RASSF3 expression demonstrated a 6.2-fold increase in RASSF3 transcripts in GH3 cells stably transfected with Lenti-RASSF3 and a 7.1-fold increase in stably transfected GT1.1 cells compared with nontransfected parental controls (Figure 4A). In contrast, RASSF3 expression was reduced by 51% in GH3 Lenti-RASSF3 shRNA transfectants and 56% in GT1.1 Lenti-RASSF3 shRNA transfectants compared with RASSF3 expression in the control cell lines (Figure 4A). Western blot analysis confirmed the increase in RASSF3 protein expression in GH3 Lenti-RASSF3 and GT1.1 Lenti-RASSF3 transfectants (Figure 5). RASSF3 protein expression was reduced in GH3 and GT1.1 Lenti-RASSF3 shRNA transfectants compared with RASSF3 expression in the control cell lines (Figure 5). Proliferation of GH3 and GT1.1 cells was examined by MTT assay 2–5 days after transfection with lentivirus containing Lenti-RASSF3, Lenti-RASSF3 shRNA, Lenti-GFP, and nontransfected cells. As shown in Figure 4B, Lenti-RASSF3 transfection suppressed GH3 and GT1.1 cell growth. In contrast, Lenti-RASSF3 shRNA transfection promoted GH3 and GT1.1 cell growth. Also, flow cytometry (Annexin V–FITC–PI) was used to study the effect of RASSF3 on apoptosis. Lenti-RASSF3 transfection promoted GH3 and GT1.1 apoptosis, while Lenti-RASSF3 shRNA transfection inhibited GH3 and GT1.1 apoptosis (Figure 4C). GH3 and GT1.1 cell growth was inhibited and apoptosis was induced by RASAF3, therefore, we further studied the effect of RASSF3 on the protein level of p53, Bax, Bcl-2, and caspase-3, which are related to apoptosis in GH3 and GT1.1 cell lines. After 72 h transfection, there was an increase in Bax, p53, and caspase-3 and a decrease in Bcl-2 in Lenti-RASSF3-transfected GH3 and GT1.1 cells (Figure 5).

### RASSF3-induced Apoptosis is p53 Dependent

**Figure 6:**
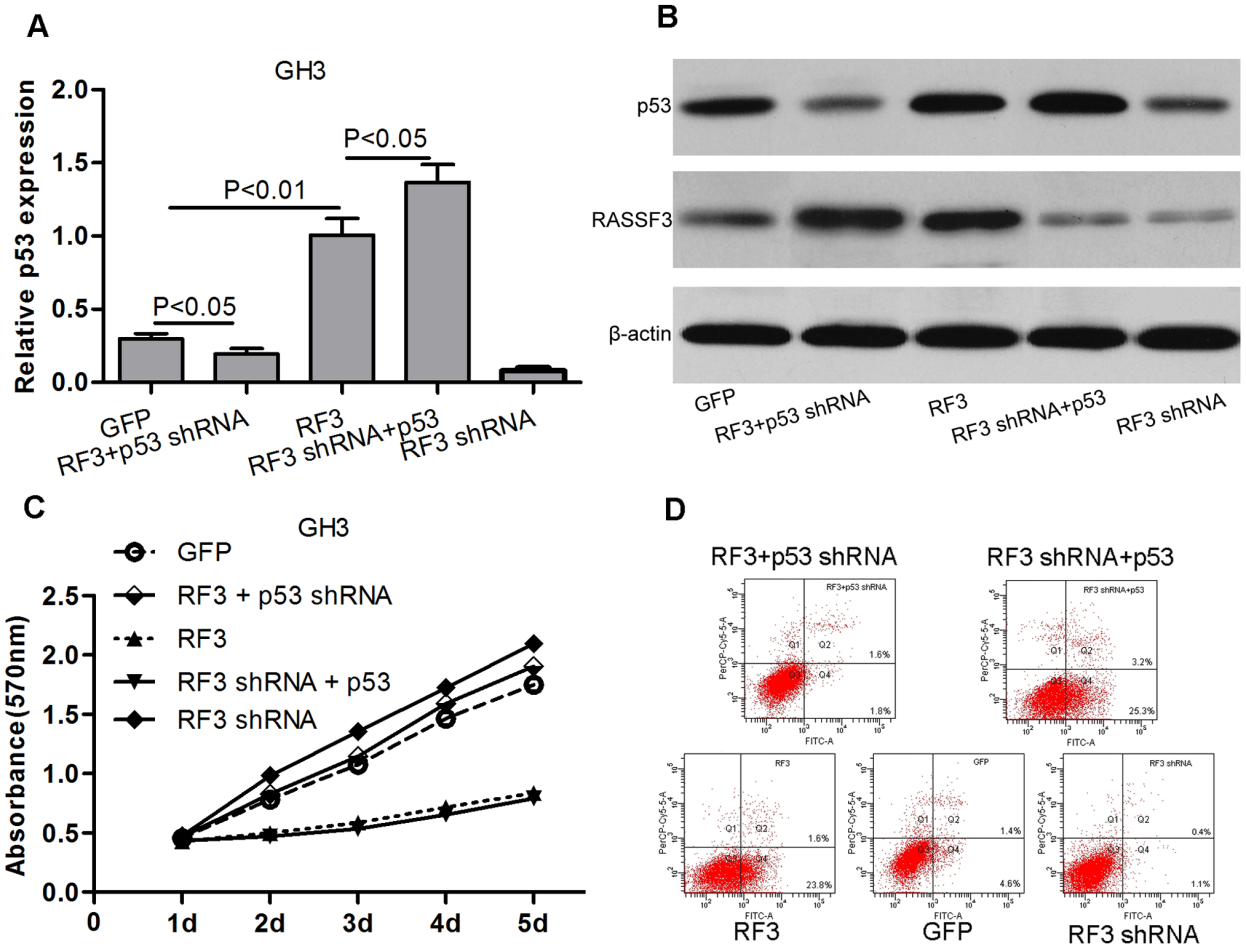
RASSF3-induced apoptosis is p53 dependent. (A) Compared with the Lenti-RASSF3-transfected GH3 cells, qRT-PCR analysis demonstrated that Lenti-P53 shRNA reduced p53 expression by 81%. Compared with Lenti- RASSF3 shRNA GH3 cells, Lenti-p53 increased p53 mRNA expression by 17.1-fold in Lenti-RASSF3 shRNA-transfected GH3 cells. (B) Western blotting analysis for RASSF3, p53. (C) Proliferation in GH3 cells was measured by MTT assay after Lenti-p53 shRNA transfection of Lenti-RASSF3-transfected GH3 cells, and Lenti-p53 transfection of Lenti-RASSF3 shRNA-transfected GH3 cells. Absorbance was read at 570 nm and the average was calculated for 3 wells. p53 suppression blocked the effect of RASSF3 on cell proliferation, and p53 overexpression showed a similar effect to RASSF3. (D) Apoptosis in GH3 cells was measured by Annexin V/PI staining following Lenti-p53 shRNA transfection of Lenti-RASSF3-transfected GH3 cells, and Lenti-p53 transfection of Lenti-RASSF3 shRNA-transfected GH3 cells. Early apoptotic cell populations were significantly decreased after Lenti-p53 shRNA transfection, and increased after Lenti-p53 transfection.

p53 is involved in apoptosis of many tumor cells, therefore, we studied whether RASSF3 promotes apoptosis through the p53 pathway. We knocked down p53 by Lenti-P53 shRNA in Lenti-RASSF3-transfected GH3 cells, and overexpressed p53 by Lenti-P53 in Lenti-GFP GH3 cells (Figure 6A and B). In RASSF3-overexpressed GH3 cells, p53 suppression restored cell growth and suppressed apoptosis (Figure 6C and D). In Lenti-RASSF3 shRNA-transfected GH3 cells, p53 overexpression inhibited proliferation and promoted apoptosis (Figure 6C and D). In addition, the effect of p53 overexpression was similar to RASSF3 overexpression. These findings demonstrate that p53 knockdown can block the antitumor effects of RASSF3, and imply that RASSF3-induced apoptosis depends on p53.

### RASSF3 Overexpression does not Change Somatotroph Adenomas Cell Invasion Ability

**Figure 7:**
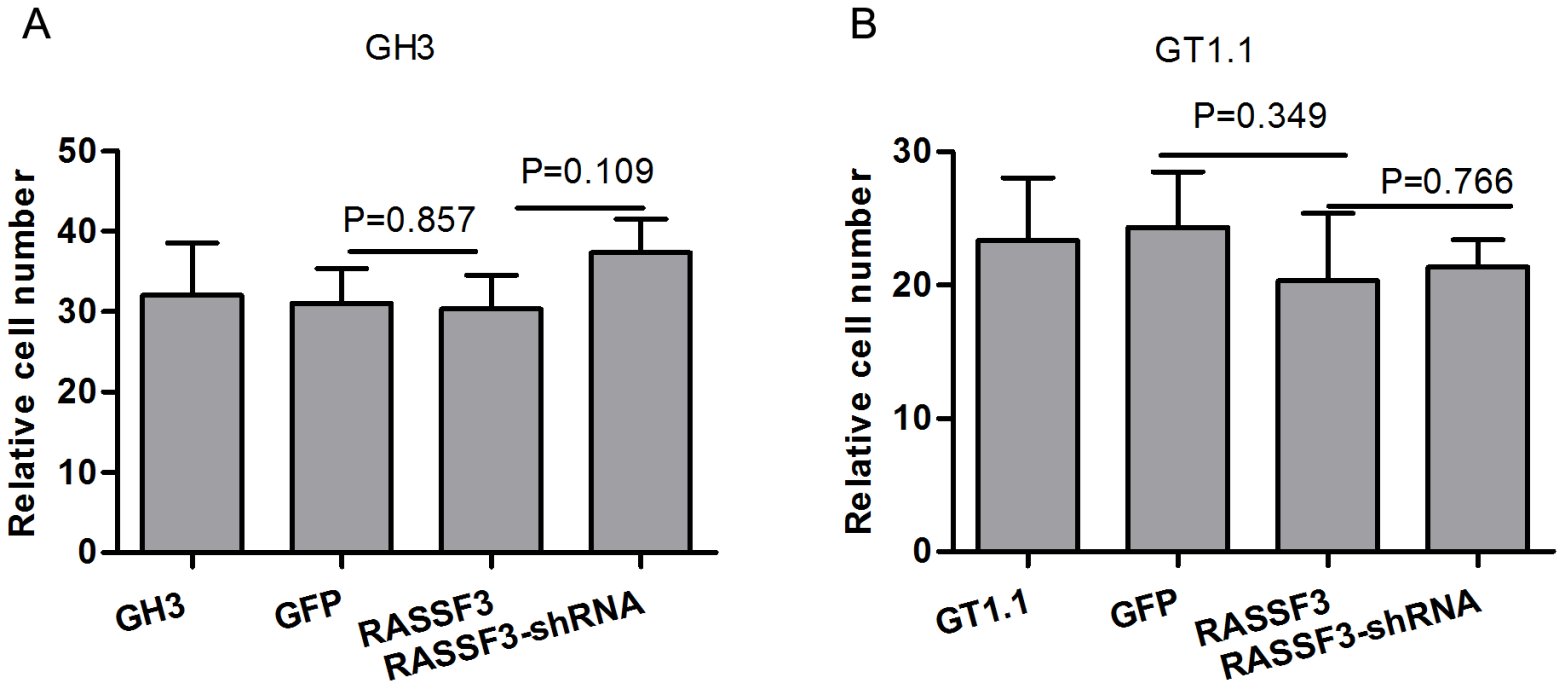
Effect of RASSF3 expression on cell invasive capacity in the Matrigel model. Matrigel assays showing the effect of Lenti-RASSF3 shRNA and Lenti-RASSF3 on the invasive potential of GH3 and GT1.1 cells. The bar graph indicates the mean number of invaded cells at 24 h after top-side cell seeding. Lenti-RASSF3 or Lenti-shRNA did not affect invasion of GH3 and GT1.1 cells.

To investigate whether RASSF3 was involved in somatotroph adenomas cell invasion, Transwell migration assay was performed. Neither RASSF3 overexpression nor silencing changed cell migration ability compared with Lenti-GFP, and nontransfected cells (Figure 7). The results suggest that RASSF3 is not associated with cell migration.

## Discussion

The pathogenesis of pituitary adenoma tumorigenesis and progression are poorly understood. Recently, epigenetic changes, especially gene promoter hypermethylation that could inhibit gene transcription has gained considerable attention as an important mechanism of silencing TSGs in tumors [28]. In pituitary adenomas, several important TSGs, are frequently inactivated by promoter hypermethylation [10]–[14]. However, very few whole-genome methylation detection strategies have been applied to the analysis of DNA methylation in pituitary adenomas. In our NimbleGen HG18 CpG Promoter Microarray study, the promoter of RASSF3 was frequently methylated in somatotroph adenomas but not in normal adenohypophyses. The RASSF3 gene contains five exons and encodes a 247-amino-acid protein (28.6 kDa) with a highly conserved Ras association (RalGDS/AF-6) domain at the C terminus [29]. RASSF3 is ubiquitously expressed in normal tissues [29], and downregulated in human lung, uterus and colon tumors [26]. RASSF3 is overexpressed in mammary gland of tumor-resistant MMTV/neu mice, and exogenous RASSF3 expression reduces cell viability and induces apoptosis in human breast cancer cell lines so, RASSF3 is considered to be responsible in part for resistance to mammary tumor development in neu transgenic mice [26]. These reports suggest that RASSF3 functions as a tumor suppressor like other RASSF proteins. However, as far as we are aware, there have been no studies about the mechanism of RASSF3 silencing or RASSF3 function in pituitary adenomas.

We next investigated RASSF3 methylation level by DNA bisulfite treatment and pyrosequencing analysis, and confirmed the results of the NimbleGen HG18 CpG Promoter Microarray study. In our studies, there was a significant correlation between RASSF3 expression and promoter methylation in human pituitary somatotroph adenomas. Because RASSF3 downregulation and promoter hypermethylation were also observed in GH3 and GT1.1 cell lines, we used these two cell lines to confirm whether methylation of RASSF3 was the reason for its silencing. We treated GT1.1 and GH3 cells with methylation inhibitor 5-Aza and histone deacetylase inhibitor TSA. RASSF3 promoter demethylation and mRNA re-expression were observed after 5-Aza treatment of the GT1.1 and GH3 cell lines. TSA treatment alone did not alter the methylation levels or restore RASSF3 expression. However, administration of TSA following 5-Aza led to a further decrease in the methylation level of RASSF3 and had a significant additive effect in restoring RASSF3 expression. The results of demethylation treatment indicate that RASSF3 expression is regulated by its gene promoter methylation status, and suggest that its silencing in somatotroph adenomas might be a result of hypermethylation. To study the effect of RASSF3 on somatotroph adenomas, we transfected RASSF3 into GH3 and GT1.1 cells. Exogenous RASSF3 expression in GH3 and GT1.1 cells suppressed growth, promoted apoptosis, accompanied by increased p53, Bax, and caspase-3 protein expression, and decreased Bcl-2 protein expression. RASSF3 suppression by Lenti-RASSF3 shRNA confirmed its function in tumor proliferation and apoptosis. p53 contributes to the apoptosis induction by RASSF1A and RASSF5 [30],[31], and p53 is involved in somatotroph adenoma senescence [32], therefore, we studied whether RASSF3 promoted apoptosis through p53. In RASSF3-overexpressed GH3 cells, p53 suppression restored growth and suppressed apoptosis. In Lenti-RASSF3 shRNA -transfected GH3 cells, p53 overexpression induced apoptosis and inhibited proliferation. The effect of p53 overexpression was similar to RASSF3. These studies demonstrate that p53 is necessary for RASSF3-induced apoptosis. Therefore, methylation-induced silencing of RASSF3 may promote tumor cell growth by inhibition of apoptosis and promotion of proliferation. Although RASSF3 promoter hypermethylation was frequent in human pituitary somatotroph adenomas, there was no significant difference in RASSF3 expression and methylation level for different tumor grades, and RASSF3 overexpression did not affect cell invasiveness of somatotroph adenomas. These results indicate that silencing of RASSF3 by promoter methylation is ubiquitous in pituitary somatotroph adenomas, and might be an early event in somatotroph adenoma tumorigenesis.

In summary, our results suggest that RASSF3 gene silencing by promoter methylation is an important early event in somatotroph adenoma tumorigenesis. Hypermethylation-induced silencing of RASSF3 may contribute to tumor cell growth by apoptosis inhibition through the p53 pathway. In the context of the data presented in this study, 5-Aza induced re-expression of RASSF3 might offer new avenues for treatment of somatotroph adenomas.
